# Evaluating a health care provider delivered intervention to reduce intimate partner violence and mitigate associated health risks: study protocol for a randomized controlled trial in Mexico City

**DOI:** 10.1186/1471-2458-14-772

**Published:** 2014-07-30

**Authors:** Kathryn L Falb, Claudia Diaz–Olavarrieta, Paola A Campos, Jimena Valades, Roosebelinda Cardenas, Giselle Carino, Jhumka Gupta

**Affiliations:** Chronic Disease Epidemiology and Social and Behavioral Sciences, Yale School of Public Health, New Haven, CT USA; Center for Interdisciplinary Research on AIDS, Yale School of Public Health, New Haven, CT USA; Population Council and National Institute of Public Health, Mexico City, MEXICO; Innovations for Poverty Action, Mexico City, MEXICO; International Planned Parenthood Federation/Western Hemisphere Region, New York, NY USA

**Keywords:** Intimate partner violence, Violence against women, Randomized controlled trial, Screening, Counseling, Mexico

## Abstract

**Background:**

Intimate partner violence (IPV) victimization is a prevalent issue among women residing in Mexico City. Comprehensive and integrated health care provider (HCP) delivered programs in clinic-settings are needed, yet few have been evaluated in Latin America, including Mexico. In addition, there has been minimal attention to interventions among lower income women presenting at settings outside of antenatal care clinics. The current randomized controlled trial seeks to increase midlevel HCPs’ capacity, specifically nurses, who are often the first point of contact in this setting, to identify women presenting at health clinics with experiences of IPV and to assist these women with health risk mitigation. Specific outcomes include changes in past-year IPV (physical and/or sexual), reproductive coercion, safety planning, use of community resources, and quality of life.

**Methods/Design:**

Forty-two public health clinics in Mexico City were randomized to treatment or control clinics. Nurses meeting eligibility criteria in treatment groups received an intensive training on screening for IPV, providing supportive referrals, and assessing for health and safety risks. Nurses meeting eligibility criteria at control clinics received the standard of care which included a one-day training focused on sensitizing staff to IPV as a health issue and referral cards to give to women. Women were screened for eligibility (currently experiencing abuse in a heterosexual relationship, 18-44 years of age, non-pregnant or in first trimester) by research assistants in private areas of waiting rooms in health clinics. Consenting women completed a baseline survey and received the study protocol for that clinic. In treatment clinics, women received the nurse delivered session at baseline and received a follow-up counseling session after three months. Surveys are conducted at baseline, three months, and fifteen months from baseline.

**Discussion:**

This study will provide important insight into whether a nurse-delivered program can assist women currently experiencing abuse in a Latin American context. Findings can be used to inform IPV programs and policies in Mexico City’s public health clinics.

**Trial registration:**

NCT01661504

## Background

Intimate partner violence (IPV) against women is a critical health, human rights, and development concern, with global estimates indicating that approximately one in three women have reported such violence at some point in their life [1]. Research has documented a range of negative health effects from IPV, including poor mental health, unwanted pregnancies, reproductive coercion, and vulnerabilities to HIV and sexually transmitted infections [2–7]. Within Mexico, approximately 25%-40% of women who receive health sector services have reported violence from their partners at some point in their life [8–10]. These lifetime IPV figures are comparable to other countries in Latin America and the Caribbean [11], and underscore the importance of interventions in health care settings. These approaches are critical as women experiencing IPV have been shown to disproportionately utilize health care services [12], and providers may be in a unique opportunity to intervene as they are among the few outside contacts a woman may have exposure to.

Despite the potential importance of health care provider (HCP)-delivered IPV interventions and in particular, HCP IPV screening programs, such approaches remain controversial [13, 14]. Much of the debate surrounding the screening of women for IPV by HCPs is due to mixed evidence regarding the effectiveness of such approaches in reducing IPV and improving health outcomes [15, 16]. Moreover, IPV screening by HCPs is not universally recommended by professional health organizations [17]. However, it remains unclear if the screening alone is an ineffective approach for addressing IPV or if HCPs do not fully adhere to screening protocols. Lack of fidelity towards consistent screening has been noted in systematic reviews due to a variety of barriers perceived by HCPs [18]. In addition, as studies evaluating screening for IPV involve vulnerable populations of abused women, there may be high levels of attrition [15], which may vary differentially by severity of IPV victimization and thus limit extrapolation of findings. However, the evidence-base is growing to suggest that comprehensive and systems-approaches to identifying and counseling women experiencing IPV in health settings is an effective strategy [19–21] to address both IPV victimization and related health concerns.

Thus, rigorous evaluations, including randomized controlled trials, of these combined and integrated HCP approaches are needed [17]. In particular, there is limited evidence of their utility in Latin American countries, such as Mexico, including attention to the potential mitigation of broader health outcomes that are consistently associated with IPV, such as quality of life. One key health outcome that may also be reduced with integrated screening and counseling programs is reproductive coercion, which may involve birth control sabotage or other pressure to become pregnant [22, 23]. In addition, while one pilot study conducted in Peru documented increases in safety planning behaviors among abused pregnant women who were screened for violence and received a counseling-based empowerment intervention, uncertainty exists regarding the effectiveness of safety planning or related counseling approaches in low and middle income countries in clinics that do not focus solely on antenatal care [24]. The examination of such broader short and long-term health outcomes within the context of HCP delivered IPV interventions has been increasingly highlighted as an important area of intervention research [16, 25].

Evaluations of the efficacy of these HCP responses to IPV are particularly needed within Mexico City, as the health system’s response to IPV has been fragmented at best. Briefly, Mexico City’s Ministry of Health (MoH) began implementing a domestic violence program in 2006. This program focused primarily on training health staff at hospitals on violence prevention and referral, in accordance with their internal protocol regarding IPV [26]. The IPV program run by the MoH operates only within select hospitals (12 out of 31); the program has staff specifically assigned to offer psychological services to victims of IPV. However, despite interest in expansion and efforts by organizations in the region to build public sector capacity to address IPV, the program has not expanded due to limited resources [27].

Additional challenges of the health sector response to IPV were noted in the planning phase of the study after in-depth discussions with the MoH and nurses at clinics. First, HCPs, hereafter nurses, at health clinics have limited to no information about where to refer IPV cases screened at the health clinics. Furthermore, nurses were given a self-efficacy test and knowledge test related to IPV as part of piloting the intervention and results showed that only 45% of nurses (n = 187) were prepared to make appropriate referrals for women experiencing IPV. Similar challenges regarding skills, referral systems and general knowledge of IPV have also been noted in other health sector responses in low and middle income countries [28, 29]. In addition, despite efforts to address IPV [30], there have been no trials in this context to assess effectiveness of health sector interventions in Mexico City. This is in contrast to the recent improvement in Mexican legislation which recognizes the right of women to live free from violence, and asks for improvements in the provision of services to the population [31].

To address these gaps in knowledge, we are conducting the currently described randomized controlled trial to assess the efficacy of a comprehensive HCP delivered intervention in Mexico City. The primary objective of the study is to increase nurses’ capacity and self-efficacy to identify IPV and assist women with risk mitigation. Through this objective, nurses will be equipped to provide counseling and information for women experiencing IPV, linking women to resources in their community, creating safety plans to reduce risk of severe violence, and to mitigate any adverse health risks. The trial outcomes are to: (1) assess the efficacy of an enhanced nurse-delivered screening and counseling program on the primary outcome past year IPV, including severe IPV (sexual or physical) and injuries from such IPV [32]; and secondary outcomes (b) reproductive coercion [23] (c) use of community-based resources and safety planning [33]; and (d) quality of life and mental health [34]; versus minimum standard of care; (2) to qualitatively examine which programmatic components may serve as mechanisms for observed quantitative changes stated in the outcomes; and (3) to synthesize study findings and (a) create recommendations for clinic-based intervention programs to address IPV in low and middle income countries and (b) disseminate information as reports, presentation, and peer-reviewed publications.

## Methods/Design

### Study design

Our study utilizes a cluster-randomized controlled trial design and will take place between 2012 - 2015. Briefly, 42 public health clinics (first level of care facilities) were randomized to either the treatment or control conditions and women are to be assessed for information at baseline, time 2 (3 month follow-up), and time 3 (15 month follow-up). Further details are described below.

### Study setting

Mexico City, the capital and most populous urban center of Mexico, provides the study setting in which our research will occur. In this megacity, almost half of the population is uninsured (approximately 3,859,963 residents) [35]. The uninsured population receives health care, through the federal health care program, Seguro Popular, at MoH clinics in Mexico City, this public insurance scheme aims at providing universal health care; a right recognized in the Mexican constitution of 1983 [36].

The MoH health system in Mexico City comprises 206 health clinics and 31 hospitals. The 206 MoH clinics are classified into Type I, Type II and Type III based on level of care. Clinics classified as Type III are large clinics that offer a broader array of services than Type I or II clinics. For instance Type III clinics offer laboratory tests. Clinics classified as Type I and II, are community clinics with 1 or 2 doctors and about 2 nurses; their focus is on community outreach and immunizations with Type I being the smallest level of community health center. Type III clinics were selected to participate in the study after conversations with the MoH because of their lack of IPV services and larger patient volume compared to Type I and Type II. These Type III clinics, chiefly located in low and middle income neighborhoods, serve primarily a low income population, and are thus eligible for the Seguro Popular. The number of women served by these health clinics varies greatly from borough to borough. Over 2,336,000 residents use the services of the MoH health clinics and hospitals annually [37].

At these Type III primary care clinics, the first points of contact for patients are nurses. The flow of patients at these clinics is as follows: patients line up early in the morning in order to get an appointment on a first come first served basis; although the MoH is currently implementing an appointment system that will reduce waiting times for patients. Nurses take somatometry measurements on all patients before they are seen by the doctors. Social workers do not have contact with every patient unless they are specifically needed in instances if patients need a referral to a hospital or to a different health clinic.

Out of the 60 Type III health clinics, one was excluded from potential recruitment because it was located in the borough of Milpa Alta; a remote and rural location with limited access to community resources and a small populace (<2% of Mexico City’s population). Two additional clinics were excluded to reduce contamination. These hospitals were located less than three blocks from hospitals and some hospitals also had violence against women programs.

### Randomization, sample size, and power analysis

Approximately 900 women from 42 health clinics were calculated to be sufficient sample size in order to achieve 80% power to detect a 15% difference in IPV frequency between treatment versus control arms at the α < 0.05 statistical significance level. A conservative intraclass correlation of 0.07 was assumed for clustering at the clinic level. This total sample size takes into account an attrition rate of 45%. The study proposal has budgeted for tracking participants that are relatively more difficult to track in order to reduce attrition.

Health clinics were first stratified by city zone (e.g. Center, North, East, West, South). Of the 57 health clinics that met inclusion criteria, 42 were randomly selected and randomized to treatment or control conditions, based on sample size calculations. To select the 42 health centers, all centers were assigned random numbers in Excel and sorted from smallest to largest; health centers were selected based on city zone and in order of their random number. Randomization was completed in STATA.

### Study population, recruitment, and retention

**Figure 1:**
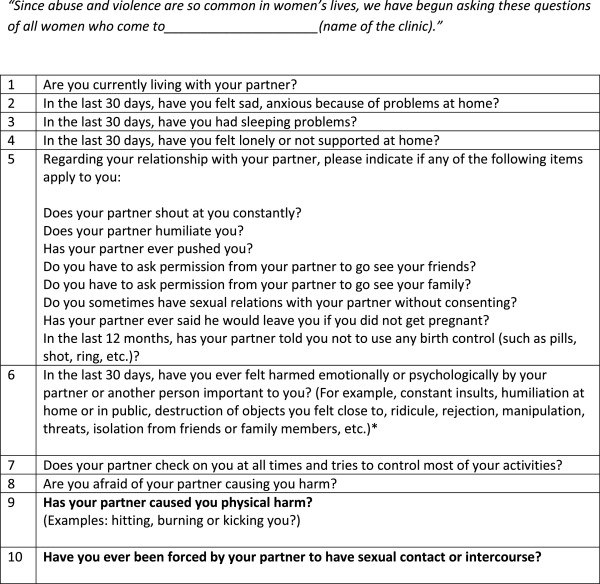
**Abuse assessment screening.**

Women were eligible to participate in the study if they were between the ages of 18-44 years, currently in a heterosexual relationship with a male partner, responded in affirmative to past year sexual or physical IPV, and was not pregnant or pregnant in their first trimester. Study exclusion criteria included cognitive impairment (e.g. slurred speech), seeking treatment for life threatening emergency care, and intending to relocate within two years. Research assistants approached women in the waiting rooms of participating clinics, verified eligibility for the study, and asked women to take an assessment screening that contained items from an abuse assessment screen that is widely used by International Planned Parenthood Federation/Western Hemisphere Region (IPPF/WHR) in Latin American and Caribbean countries and has been previously used in studies occurring in Mexico City [38–40]. Based on feedback from piloting the assessment, the final tool consisted of eleven questions in order to build rapport between the research assistant and participant. The first nine questions were in regards to the woman’s health and relationship with her partner, including emotional abuse, as directly asking about physical or sexual IPV at the very beginning of the assessment was rendered as too sensitive during our piloting phase. Based on feedback from focus groups carried out with IPV survivors at a community domestic violence agency, concrete examples of physical and sexual IPV were included in the questions (see Figure 1). If a woman answered at least one affirmative response to the validated screening items on physical and sexual IPV contained in the assessment, they were invited to participate in the study; those interested completed written and/or verbal informed consent. Both the assessment and the informed consent process took place within a private area of the clinic.

At baseline data collection, women were asked for their contact information, including the names of three other people in case the woman could not be reached for follow-up appointments. Research assistants called every number provided by the participant to verify that the phone numbers were functional. Participants received a call on average 3 days after they completed the baseline to verify contact information. All participants agreed that it was okay to call them and research assistants were trained to ask if it was a safe time to talk and did not mention violence or the study if another person answered the phone.

All women received monthly follow-up phone calls to remind them of 3 month and 15 month follow-up appointments. At all times their disclosure of IPV remained confidential and the purpose of the study was not be shared with anyone. Upon conclusion of the surveys and counseling at baseline, all women received a snack for their time. At the three month follow-up they received a gift card for $15 USD and at the fifteen month follow-up participants will receive $20 USD. The amounts and type of compensation were determined based on consultation with Mexfam (Mexican Foundation for Family Planning) and the MoH, as well as responses from a short survey given to women at health clinics during our piloting phase regarding their preferred mode of compensation (e.g. phone credit, food, gift card, *etc*).

Data were collected through a computer-assisted survey which has been shown to improve response rates to sensitive behaviors [41]. Participants completed the survey in a private space in the clinic and were able to listen to the questions in Spanish through headphones and answer on the keyboard. Research assistants were available during this period in case the participant had any questions. Given that clinics were randomized and not individuals, research assistants were not blinded to whether they were in a treatment or control clinic.

### Intervention description

Women who participated in the study at treatment clinics received the following intervention: (1) integrated IPV and health screening; (2) supportive care; (3) safety planning and harm reduction counseling; (4) supportive referrals; and (5) booster counseling sessions at three months post baseline. The majority of materials utilized for the intervention were adapted from existing IPPF/WHR materials used in other Latin American and Caribbean contexts and were adapted to the characteristics of Mexico City [38]. A referral directory was also created with the contact information of local community agencies that provide services to IPV victims. During the development phase of the referral directory, IPV staff at the MoH suggested that only community agencies providing free services be included. All community agencies listed in the directory were visited by research staff and addresses and phone numbers were verified. Furthermore, for each community agency, research staff obtained the name of a specific contact person to include in the referral directory. Contact names were included in the referral directory based on pilot focus group research with IPV survivors enrolled at a community agency. Pilot work revealed that women felt more comfortable asking for services if there was a specific person to contact at an agency; and that only having the name of an agency was a deterrent.

Of the agencies, two programs were highlighted: (1) SEPAVIGE, the MoH IPV program, (Gender based Violence Special Services) and UAVIF (Family Violence Prevention and Care Unit). There are approximately 14 SEPAVIGE units located primarily within hospitals. Key services include free psychological treatment and group therapy to IPV victims. UAVIF is associated with the Ministry of Social Development and it consists of 16 units in each district of Mexico City. Each unit offers legal and psychological services. They are also in charge of identifying women eligible for an IPV conditional cash transfer program. The referral directory was handed out to nurses during the training Details are presented in Table 1.

**Table 1:** **Intervention components**

Intervention component	Description
Integrated IPV and health screening	Women will be screened for IPV including emotional, physical, and sexual violence, as part of a general health assesment.
Supportive care	Nurses will be trained to provide non-judgmental and empathetic counseling, .
Safety planning and harm reduction counseling	Nurses will discuss safety planning measures with women, including escape routes or places of refuge, packing and storing a bag with important belongings, memorizing phone numbers, talking to children about what to do in cases of violence, and staying away from rooms with weapons. Harm reduction counseling will include the partners’ use of alcohol and illicit drugs, how to remove weapons, options for protecting reproductive health, such as protecting against unplanned pregnancy, sexually transmitted infections, and other individual-specific health risks.
Supportive referrals	Women will be counseled about local IPV and sexual assault resources according to their specific needs. Access and utilization will be facilitated by contacting programs together or by offering the woman step-by-step directions. Business-sized referral cards which contain contact information for local resources, will be given to all women.
Booster counseling sessions at 3 months	Components of above screening, referral, safety planning and harm reduction will be reviewed and redelivered to program participants. Sessions will occur in clinic and information will be recorded in patient charts.

Nurses from all 42 health clinics were invited to participate in the training based on the following criteria: morning shift nurses (due to the walk-in basis of appointments and afternoon shifts not occurring at all clinics) and not a field nurse (due to their limited time at the health clinics). A total of 197 nurses (approximately 8% male nurses and 45% in treatment clinics) received the training. 147 nurses are actively participating in the study (49% in treatment clinics) (26% staff dropped out primarily due to staff turnover at clinics). On average, 4 nurses were trained at each health clinic. Nurses in the intervention group received a 3 day training that covered all the topics of the intervention and then nurses received up to 3 visits by research staff to practice delivering the intervention through role – playing exercises individually and in the clinics. In-depth training was needed as nurses did not feel very comfortable talking about IPV and several rounds of role playing exercises were needed for nurses to gain confidence. Role-playing exercises with research staff were chosen as it preserved rapport between nurses and the research team as well as increased confidentiality of nurses requiring additional training. All training activities were conducted by the research team, IPPF training consultants, and invited guests from local organizations and government offices who specialize in this area. Specific topics in the training included an introduction to IPV, health consequences of IPV, legal considerations in Mexico City, methods to screen for violence and assess for health implications such as reproductive coercion, safety and ethical considerations, referral methods and linkages to other organizations in the community. An educational video modeling the counseling sessions was also developed by the study team for the training of treatment nurses. This video was shown and discussed during research assistant visits to the health clinics as part of the training.

Women in the control clinics were given a referral card containing general information on IPV and a list of resources, which was consistent with the current goal for standard of care in the Mexico City MoH. The referral card is the size of a business-card so that it may easily be hidden from male partners, and is widely used in other IPV screening programs [42]. Staff in control clinics received a one day training that focused mainly on sensitizing nurses and training them on using the abuse assessment. Control clinic staff will receive the intervention training upon completion of the study.

### Ethics

Conducting research with women who have recently experienced violence represents a number of safety concerns [43]. Ethical procedures were guided by World Health Organization standards for conducting research on domestic violence [44] and were taken into account during study preparations. First, all women will provide informed consent which stresses the voluntary nature of the study, the ability to skip questions as necessary, confidentiality, and outlines potential risks and benefits of participation. At the end of the survey, health care providers explain available resources for emotional support and provide a list of resources. Second, the study was designed so as not to reveal to the partner the purpose of the study or the woman’s participation in the study. At all times a woman’s disclosure of IPV remained confidential, including the three friends or family members she listed in case she could not be contacted at follow-up. During follow-up calls, the specific purpose of the study was not given; rather, it was referred to as a study on “women’s health and families”. All research staff were trained by senior study personnel on study ethics. Regular de-briefings, including recommendations for self-care, occurred with research assistants to reduce vicarious trauma among staff. Research staff and nurses participating in the study were encouraged to report any emotional distress, including if they were struggling with experiences of victimization in their own lives, to principal investigators. Study procedures have been approved by the Yale School of Public Health (Protocol # 1202009793), Innovations for Poverty Action (Protocol # 555.23May-001), and National Institute of Public Health (Mexico) (Project #1089) institutional review boards.

### Quantitative assessments & analytic plan

Our primary outcome is past-year IPV [32] and our secondary outcomes include past-year reproductive coercion [7, 23], use of community-based resources [33], safety planning measures [33], and quality of life [34]. Descriptive statistics will be used to assess achievement of Aim 1. For the remaining aims, the follow analyses plans will be undertaken: (1) assess if randomization of health clinics was successful by examining if there are statistically significant differences between women’s demographics, by treatment arm, through chi-square or t-tests; (2) assess for differential loss to follow up by demographics and baseline IPV severity; (3) assess if IPV and secondary outcomes (reproductive coercion, safety planning, use of community resources, quality of life) differed at baseline, by treatment arm; and (4) conduct multilevel analyses of the effect of the intervention on outcomes utilizing appropriate models that will account for potential clustering for repeat measures, time, and at the clinic level. We will subsequently estimate adjusted models if any demographics significantly varied at baseline. Analyses will first be undertaken using the intent-to-treat approach. Following this, a per protocol analysis will be undertaken to determine whether effectiveness varied by adherence (i.e. attending the three month booster counseling session) and potentially nurse adherence.

### Qualitative assessments & analytic plan

During endline data collection, research assistants will conduct 45 qualitative interviews with female participants to provide insight into observed quantitative results. In-depth interviews will consist of questions regarding perceptions of IPV, safety planning behaviors, reproductive autonomy, and quality of life. Underscoring all questions will be the woman’s perception of how, if at all, participating in the intervention created or affected these changes. Additionally, fifteen interviews were conducted with nurses at treatment clinics to understand their experiences with the HCP delivered approach between the three month follow-up period and the final endline at fifteen months. Interviews will last no longer than one hour and be conducted in Spanish. Upon transcription of audio recordings, interviews will be translated into English. All interviews will be coded using inductive thematic analysis [45] through which emerging themes will be discussed with principal investigators.

### Process evaluation

Throughout the study period, a process evaluation is conducted, consisting of three key activities: (1) pre-post tests of the nurse training; (2) fidelity assessments with mock clients; and (3) exit interviews with women participating in the study. First, during the training of nurses, we conducted a pre-post test before and after the training to assess the change in knowledge, attitudes, and skills among nurses in regards to screening and referral for IPV. In addition to assessing nurses’ skills for addressing IPV, these pre-posttests were undertaken due to previously noted challenges that may hinder effective interventions, such as health care providers’ victim-blaming attitudes and misconceptions of IPV [28]. Throughout the study, nurses are asked to fill out the short survey at three month intervals to assess any changes, although test-retest bias is a limitation of this process evaluation method.

Second, anonymous mock clients are used in order to assess nurses’ adherence to protocols and to serve as an opportunity to provide feedback during the training phase. Existing literature has also underscored the lack of feedback given to health care providers as a reason for infidelity to screening protocols [18]. Therefore, after completing the training, all intervention nurses are visited by trained research staff. The staff member screens positive for IPV, and goes through the clinic visit, including screening, referral, and counseling following a scripted scenario. Following the health visit, the staff member completes a fidelity checklist to determine areas in which the nurse is exceeding expectations and areas in which the nurse needs improvement. All nurses are informed that they will be visited by a mock client and that all mock clients will adhere to the script used in the training. No health nurse receives more than two mock client visits throughout the study period. Feedback was given during on-site training prior to the launch of the baseline; subsequent visits were used for fidelity monitoring only. Nurses in the control group also received one visit from the mock client, but feedback will only center on standard of care, rather than comprehensive screening and counseling women. Fidelity checklists from these mock clients do not contain identifying information and were entered into a secure database upon completion. Tape recording counseling sessions were also considered as a method of assessing fidelity. However, due to safety concerns for the women, mock clients were chosen as the more appropriate method to assess intervention fidelity.

The final activity to monitor fidelity was exit interviews with women in both treatment and control clinics. Women who were participating in the study in treatment clinics were randomly selected by research staff in the clinic after they had received the intervention, based on selection of questionnaire identification numbers. Exit interviews are designed to last between 5-10 minutes where all women were asked to provide an additional written informed consent prior to completing the survey. All women in the intervention and control groups were eligible to participate in the exit interviews.

### Costing analysis

A costing analysis is also included in the study protocol. Trained research assistants have been identifying human, financial, physical, and time resources required for the HCP delivered program throughout the study. Data has been collected and categorized by research-related costs or operational costs in order to calculate the monetary value of resources needed to conduct the intervention. The costs and effectiveness of this approach will be compared between treatment versus control groups and will mirror methodology from a previous cost-effectiveness analysis of an IPV intervention [46]. This costing exercise will provide insight into the scalability of the intervention and economic impacts of broader policy recommendations.

## Discussion

This study seeks to train and improve nurses’ capacities to deliver an intervention for women currently experiencing IPV that present at health clinics in Mexico City. If found efficacious, this study may have an important influence on programming and policy for the health sector responses in city-based clinics serving low-income women in the region. Findings may also contribute to the evidence-base for comprehensive prevention strategies to reduce IPV and to respond to the needs of survivors. This intervention may be an effective health-sector tool to reduce IPV and mitigate negative effects, in addition to many other approaches including changes in the legal system or other social change approaches that focus on gender equality and the engagement of men to reduce violence [47–49].

Despite the strong study design to assess the efficacy of the HCP delivered intervention on outcomes, limitations and potential problems have been considered. As with other randomized controlled trials, participant drop-out and differential attrition is a concern [19]. To address potential problems, we have added an exclusionary criterion that women do not plan on relocating within the study period. At the time of enrollment, we also verify contact information for the woman and obtain information for three additional contacts that would know how to get in touch with her. In the analysis plan, we will assess for differential attrition and potentially adjust final models for variables that may have statistically differed between treatment arms. Other approaches may also be used to handle missing data, including multiple imputation, which has been used in other studies examining IPV screening effectiveness [15].

In addition, staff at referral community agencies that are aware of and engaged with the study may also turnover, which may have a bearing on the quality of service provision. As noted in literature regarding health sector responses to IPV, linkages to community agencies may be a critical component of success [29]. While there are agencies for referral in Mexico City, including UAVIF, the quality of services received at referral community agencies remains unclear as it is outside the scope of the current study. In addition, there may be challenges in the capacity of referral agencies to respond to significant increases in referrals. Nonetheless, we will be able to assess utilization of agency services through the quantitative assessments with participants and gain insight into experiences through qualitative interviews. Within the clinics themselves, pilot work revealed the lack of private spaces as a physical challenge of conducting the survey and administering the HCP delivered intervention. While the study team has worked with clinic staff to identify private spaces to maintain confidentiality of participants, considerations of space must be addressed if the intervention were to be scaled up. Similar challenges have been noted elsewhere [28, 29] among health clinic interventions in low and middle income countries.

Another potential limitations that may affect our ability to see changes in IPV, that result from the intervention, is the limited follow-up time of the intervention. IPV, as noted by others [50], is a complex issue that may often require many attempts to leave an abusive partner. Thus, changes in IPV may be difficult to discern given the twelve-month follow-up after the second counseling session. However, our selection of secondary outcomes including changes in use of community resources and safety planning behaviors will allow us to see potential changes that may occur on the pathway to eventually leaving a partner. Nonetheless, statistically significant changes in other secondary outcomes, such as quality of life, may also be difficult to discern in the short follow-up period. For instance, housing relocations, childcare considerations, *etc.* may also have a negative influence on quantitative measures of quality of life as a result of leaving an abusive partner. However, qualitative interviews among women at the follow-up period will provide a meaningful narrative of women’s intervention-related experiences and attempts to leave their abusive partner. The mixed methods approach to understanding intervention impacts may be important, particularly for quality of life, as previous studies have documented null associations between quality of life and IPV screening [51].

In addition, due to logistical constraints, we could not train all personnel at health clinics, thus this is not a system-wide intervention. Therefore, we are limited concerning inferences we can make regarding institutional changes necessary for comprehensive health sector responses to IPV. MoH clinics were not able to send all nurses to the training even when trainings on different dates were held, nor were we able to train all front-line staff at the clinics. Consequently, we focused our efforts on recruiting non-field based nurses who have the highest probability of seeing the most patients in the clinic and were on morning shifts. Nonetheless, we will be able to test the potential efficacy of an HCP delivered program on IPV and related outcomes in an urban, middle-income country health system setting. If proven to be a feasible approach, future studies and monitoring efforts could be built into scale-up of activities among all nurses at health clinics. This first step is particularly feasible given the MoH’s investment in IPV programs. In addition, our ongoing process evaluations and training to nurses will provide a supportive environment to increase their efficacy to address IPV.

In summary, this trial will seek to train and increase capacity of nurses in Mexico City to identify and respond to IPV and generate evidence if such an approach is effective in reducing IPV and mitigation of related health concerns. If found to be efficacious, the study may offer important contributions and insights regarding how to increase the capacity of the health sector to respond to survivors of IPV in Mexico City. Ultimately, the results of this trial will contribute to global knowledge on the key role of HCPs in addressing IPV.

### Trial status

Study recruitment is currently underway at the time of initial manuscript submission. Upon resubmission, the baseline and three-month follow-up surveys have been completed.

## Abbreviations

HCP: Health care provider
IPPF/WHR: International Planned Parenthood Federation/Western Hemisphere Region
IPV: Intimate partner violence
MoH: Ministry of Health.
